# Atypical Presentation of Spontaneous Coronary Artery Dissection

**DOI:** 10.7759/cureus.9543

**Published:** 2020-08-03

**Authors:** Chidi D Okoroafor, Abeera Akram, Annie Ong

**Affiliations:** 1 Internal Medicine, Saint Mary's Hospital, Waterbury, USA

**Keywords:** atypical spontaneous coronary artery dissection, coronary artery dissection, spontaneous coronary dissection, : acute coronary syndrome

## Abstract

Spontaneous coronary artery dissection (SCAD) is a rare cause of acute coronary syndrome, more common in young women without risk factors for, or a history of, coronary artery disease and usually occurs in the peripartum period. There are two types of SCAD: atherosclerotic and nonatherosclerotic coronary artery dissection. Management options include conservative management, percutaneous coronary intervention, or surgical intervention, depending on the presentation and extent of the dissection. We present reports of two cases of SCAD (one man and one woman) presenting to the emergency department of our community hospital in February 2020 with nonspecific symptoms.

## Introduction

Acute coronary syndrome (ACS) is one of the leading causes of presentation to the emergency department (ED) for chest pain. Many patients with ACS at presentation may have myocardial infarction. . Spontaneous coronary artery dissection (SCAD) is a rare cause of ACS, and chest pain is usually the most common presentation [1]. We present the cases of two patients (one man, one woman) with SCAD who presented to the ED of our community hospital in February 2020 with nonspecific symptoms.

## Case presentation

Case 1

A 48-year-old obese man who is a current smoker with a history of chronic obstructive pulmonary disease (COPD) presented with shortness of breath and productive cough to the ED. At presentation, he was afebrile, tachycardic (heart rate, 120 beats/minute [bpm]), tachypneic (respiratory rate, 35 breaths/minute), and hypoxic (oxygen saturation, 79% on room air). Jugular venous distention was difficult to appreciate. He had bilateral wheezing throughout the lung fields and 1+ pitting edema in both lower limbs. He was put on 5 L oxygen via nasal cannula, given intravenous (IV) steroids, albuterol nebulization, IV furosemide, and doxycycline. His arterial blood gas showed pH of 7.31, partial pressure of CO_2 _of 59 mmHg, partial pressure of O_2_ of 57 mmHg, and a bicarbonate of 26 mmol/L. He was started on bilevel positive airway pressure (BiPAP) at 50% oxygen with an inspiratory positive airway pressure of 12 and an expiratory positive airway pressure of 5 in the setting of severe hypercapnic hypoxic respiratory failure secondary to COPD exacerbation. His health care team was concerned for acute heart failure; however, the patient’s history was not significant for cardiac events.

**Figure 1:**
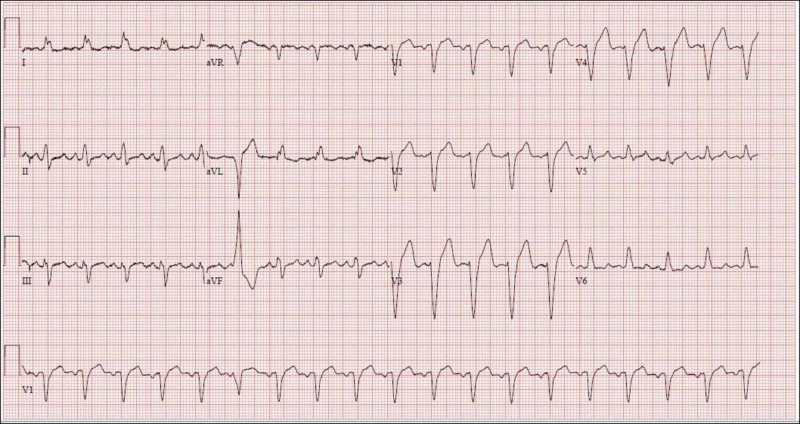
Case 1 electrocardiogram at presentation showing a normal sinus rhythm with a new left bundle branch block

**Figure 2:**
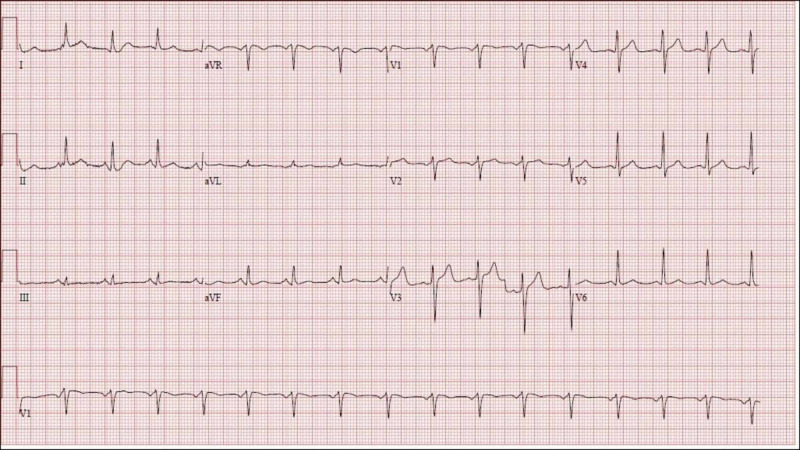
Case 1 baseline electrocardiogram six weeks prior to presentation showing a normal sinus rhythm.

He was admitted to the telemetry monitoring unit and continued BiPAP support with ipratropium/albuterol combination nebulization. The patient denied chest pain, but his electrocardiogram (ECG) showed a new-onset left bundle branch block (Figure 1) as compared to his ECG from six weeks prior (Figure 2). His troponin levels were 0.04 ng/ml at presentation, 0.04 ng/ml 2 hours later and <0.03 ng/ml after 12 hours. 

**Figure 3:**
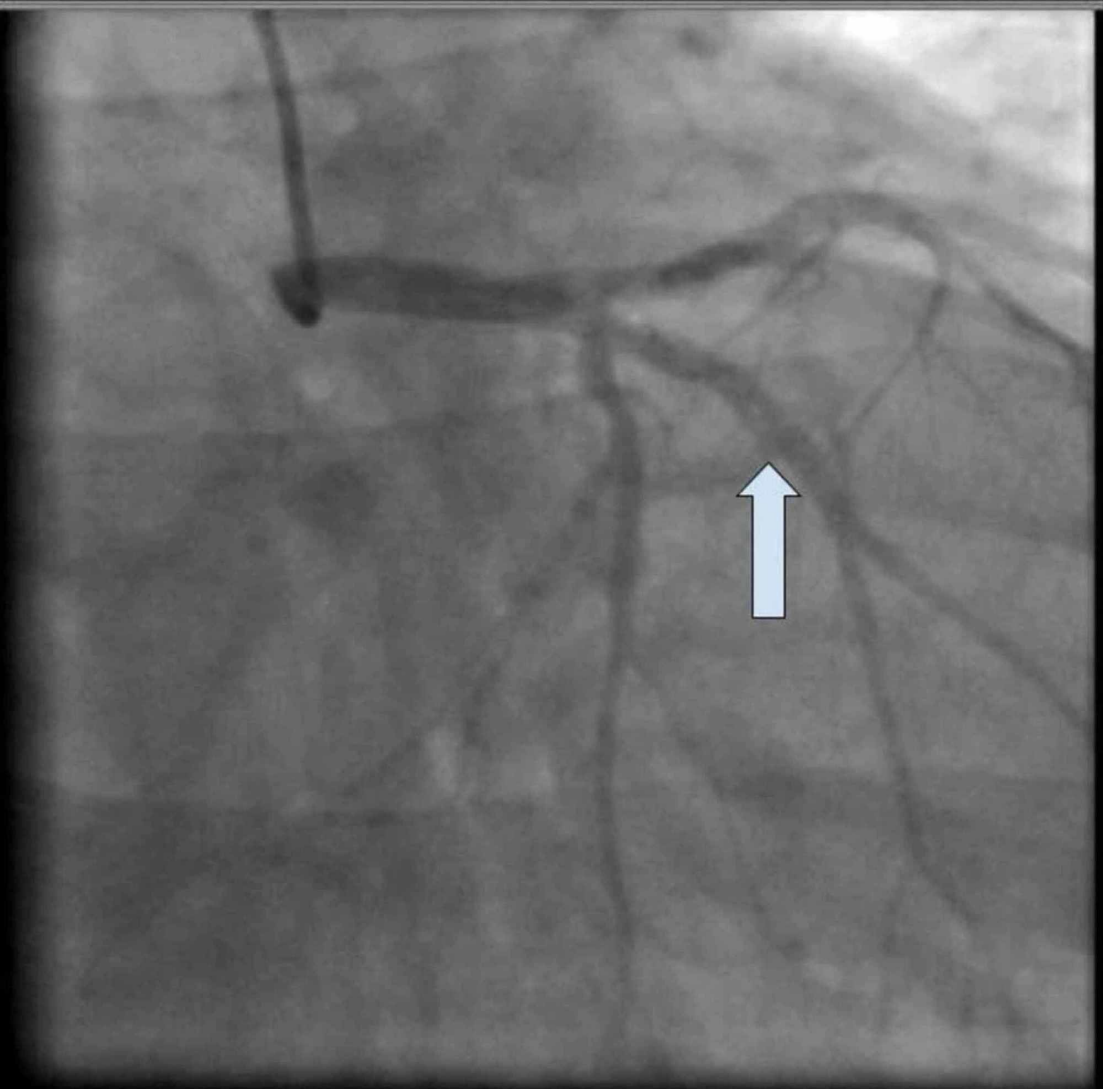
Case 1: Type 1 dissection of the first obtuse marginal artery.

A transthoracic echocardiogram showed severe left ventricular dysfunction with global hypokinesis and an ejection fraction (EF) of 30% to 35%. Cardiac catheterization showed nonischemic cardiomyopathy and SCAD of the first obtuse marginal artery, which is a type 1 dissection (Figure 3). He had thrombolysis in myocardial infarction (TIMI) score of 3 but no chest pain. Given the patient’s history of poor follow-up with outpatient care, the patient was managed medically and percutaneous coronary intervention (PCI) was not performed. He was started on aspirin, clopidogrel, sacubitril/valsartan, furosemide, and atorvastatin. He was advised to have close follow-up with a cardiologist. 

Case 2

**Figure 4:**
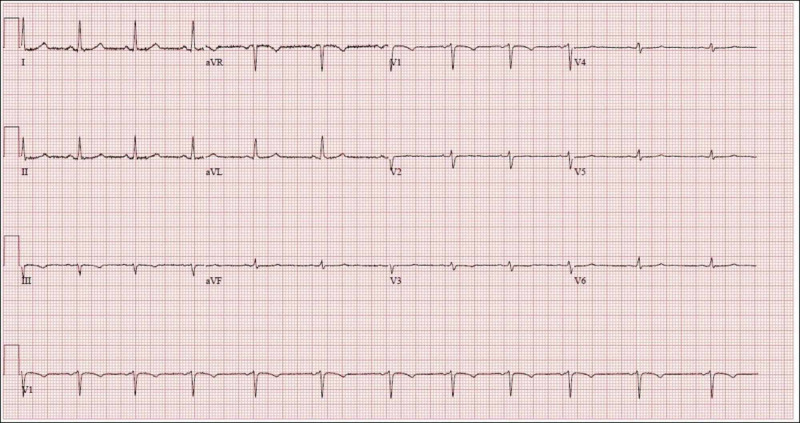
Case 2 electrocardiogram at presentation showing a normal sinus rhythm. It also meets criteria for low voltage in the precordial leads but that is not the scope of this presentation.

A nonsmoking, obese, active 38-year-old woman presented to the ED with recurrent episodes of heartburn following a meal. On the day of presentation, her heartburn was persistent, more severe, and was not relieved by calcium carbonate tablets as the symptom normally did. At presentation, she was afebrile, with a heart rate of 71 bpm, a respiratory rate of 16 breaths per minute, a blood pressure of 130/78 mmHg, and oxygen saturation of 99% on room air. She had no signs of poor perfusion or volume overload. Her ECG was normal (Figure 4); however, her troponin was elevated at 0.20 ng/ml and trended up to a peak of 2.03 ng/ml. Her transthoracic echocardiography was only significant for mild concentric left ventricular hypertrophy with an EF of 55% to 65%.

**Figure 5:**
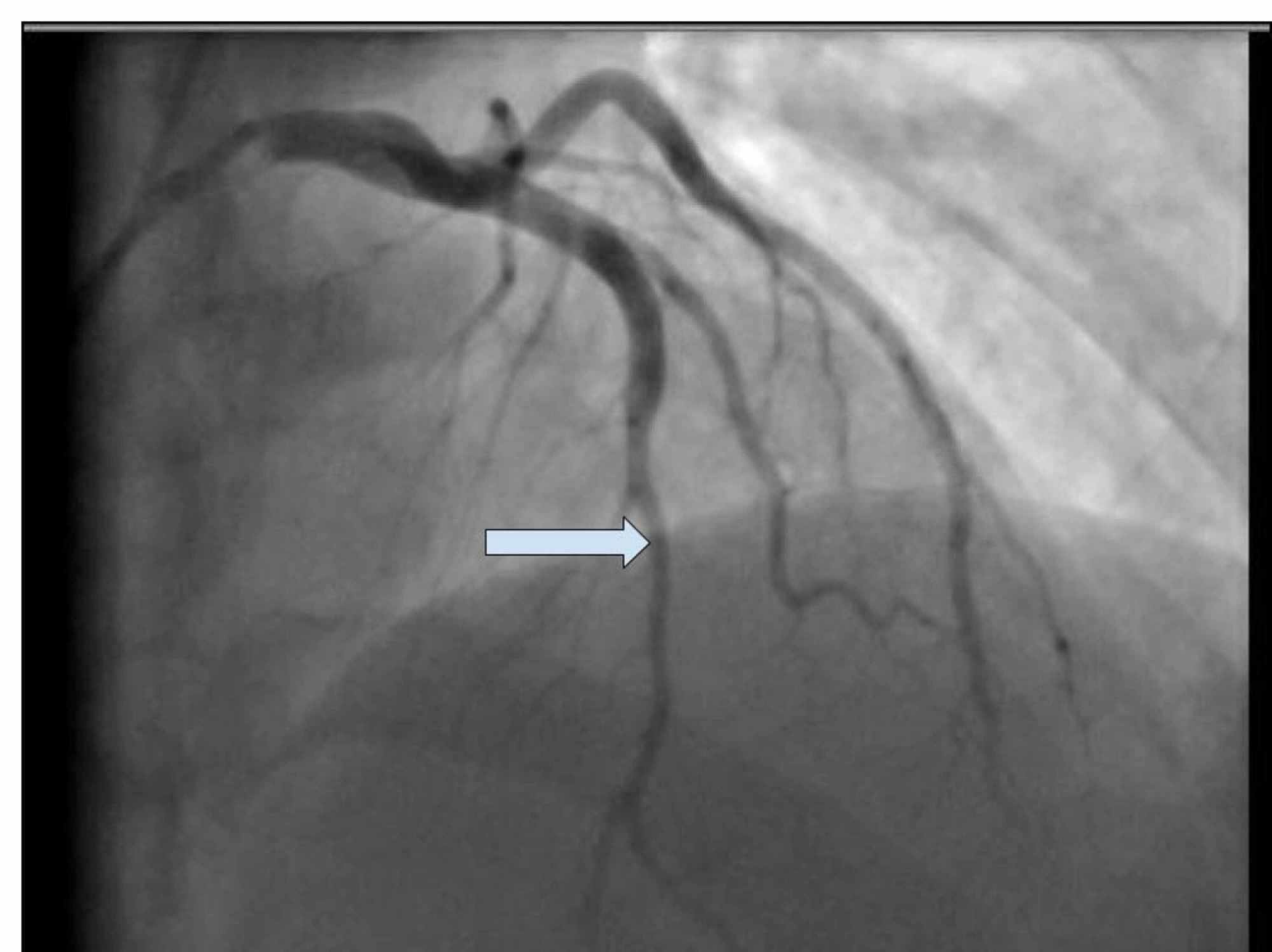
Case 2: Type 2 dissection of the distal left anterior descending.

Cardiology was consulted because of the patient’s troponin elevation in the setting of atypical chest pain. Cardiac catheterization revealed SCAD of the distal left anterior descending artery, which is a type 2 dissection (Figure 5). Given her TIMI-3 flow and absence of typical chest pain, we decided to treat her medically. She was started on metoprolol, aspirin, and atorvastatin. Given her young age and family history of early death due to brain aneurysm, we conducted further workup for vasculitis, autosomal dominant polycystic kidney disease, and fibromuscular dysplasia (FMD) via bilateral renal ultrasound, computed tomography angiography of the chest and abdomen, brain magnetic resonance imaging and magnetic resonance angiography, and tests for anti-neutrophilic cytoplasmic autoantibody-associated vasculitis, the results of which were all unremarkable. She was discharged on metoprolol, aspirin, and atorvastatin with a scheduled follow-up evaluation with a cardiologist.

## Discussion

SCAD is a nontraumatic and noniatrogenic separation of the coronary arterial wall and is a rare cause of acute myocardial infarction and ACS, accounting for 0.1% to 0.4% of ACS cases [1]. SCAD is more common in young women in the peripartum period, usually without cardiovascular risk factors; in men of any age, SCAD accounts for only 10% to 15 % of cases of ACS. Also, for men, atherosclerotic SCAD is more common than nonatherosclerotic SCAD [2]. There is some association of SCAD in men related to exertional stress, anxiety, and emotional stress [3].

Weakening of the arterial wall due to hormonal changes, cystic medial necrosis, and proteolytic enzymes from periadventitial eosinophils in susceptible patients can lead to an underlying vulnerable coronary wall [4]. Underlying factors and conditions that could potentially predispose a patient to SCAD are FMD, postpartum status, multiparity more than four births, connective tissue disorders, systemic inflammatory conditions, and hormonal therapy [5]. Stressors such as intense exercise or emotional stress, labor and delivery, intense Valsalva-type activities, and recreational drug use can precipitate SCAD [3]. SCAD can present with a variety of features including arrhythmias, unstable angina, acute myocardial infarction, cardiogenic shock, pericardial effusion and tamponade, and even sudden cardiac death [3,6-8].

Atherosclerotic SCAD is a mechanistically distinct variant of SCAD and is typically limited in extent by medial atrophy and scarring associated with atherosclerotic cardiovascular disease. Unlike atherosclerotic SCAD, nonatherosclerotic SCAD can result in extensive dissection lengths, especially in the presence of arterial fragility from predisposing arteriopathies [9]. 

The exact mechanism of nonartherosclerotic SCAD is poorly understood. An intimal tear or bleeding of the vasa vasorum with intramedial hemorrhage has been proposed, with both processes leading to the creation of a false lumen [4]. Pressure-driven expansion of the false lumen induces axial propagation of the intramural hematoma, luminal encroachment, and ultimately myocardial ischemia and infarction. 

Chest pain is the most common symptom presentation in >96% of cases; less common symptoms include arm pain, neck pain, nausea or vomiting, diaphoresis, dyspnea, and back pain [1,6,10]. In most patients, in the absence of prior trauma, the diagnosis of SCAD is made at the time of coronary angiography. 

The criteria for the angiographic definition of SCAD include the presence of a noniatrogenic dissection plane in the absence of coronary atherosclerosis, with typical changes of radiolucent intimal flap and contrast staining. An angiographic series has shown that such stereotypical changes were seen in only less than 30% of nonatherosclerotic SCAD cases [5,11]. The majority of SCAD had long and diffuse narrowing on angiography due to intramural hematoma, and this appearance was frequently unrecognized on angiography leading to underdiagnosis of this condition.

Type 1 dissection: Pathognomonic contrast dye staining of arterial wall with multiple radiolucent lumen, with or without the presence of dye hang-up or slow contrast clearing.

Type 2 dissection: Diffuse long and smooth stenosis that can vary in severity from mild stenosis to complete occlusion.

Type 3 dissection: Mimics atherosclerosis with focal or tubular stenosis and requiring optical coherence tomography (OCT) or intravascular ultrasound (IVUS) to differentiate the cause.

In patients for whom the diagnosis is considered but not secured with coronary angiography, intracoronary imaging with OCT or IVUS may be helpful. With these imaging modalities, SCAD diagnosis is made with the presence of intramural hematoma and/or a double lumen. Alternatively, repeat coronary angiography may be pursued four to six weeks later to evaluate for spontaneous angiographic healing of the dissected segment, if the diagnosis is uncertain.

Irrespective of dissection type in most SCAD patients, conservative management with medical therapy is the preferred strategy as was the case with our patients. PCI can be used for patients needing emergent revascularization [3]. Other treatment options include coronary artery bypass grafting, fibrinolytic therapy (with or without subsequent PCI), mechanical hemodynamic support, and cardiac transplantation, the selection of which should be based on patient presentation and unique characteristics [5].

## Conclusions

SCAD as a rare cause of ACS has been shown to be associated with various presentations. Some of which include anxiety, emotional stress ,exertional stress, and intense Valsalva-type activities usually with associated chest pain. In most patients, conservative management with medical therapy is the preferred approach to management. Our case series highlights the need to also consider the diagnosis of SCAD as a cause of ACS in young patients presenting to the ED with nonspecific symptoms, such as shortness of breath and heartburn, and not just those with the more typical presentation of chest pain. 
